# Mixed shock: from pathophysiology to clinical practice

**DOI:** 10.47487/apcyccv.v7i1.567

**Published:** 2026-03-28

**Authors:** Arturo Miguel Cagide, Ignacio Martín Bluro, Aníbal Martín Arias, María Natalia Pellegrini, Juan María Iroulart

**Affiliations:** 1 Servicio de Cardiología, Hospital Italiano de Buenos Aires, Ciudad Autónoma de Buenos Aires, Argentina. Servicio de Cardiología Hospital Italiano de Buenos Aires Ciudad Autónoma de Buenos Aires Argentina

**Keywords:** Shock, Vasodilation, Heart Failure, Shock, Vasodilatación, Insuficiencia Cardíaca

## Abstract

Mixed shock represents a complex haemodynamic entity arising from a variable combination of impaired contractility and vasoplegia. Its clinical recognition is challenging, as it may appear as an intermediate state in the evolution of cardiogenic or distributive shock, or result from the simultaneous coexistence of distinct pathophysiological mechanisms. Neurohormonal activation, which is common across both ends of the shock spectrum, contributes to fluid retention and further deterioration of circulatory function. Although pathophysiological interpretation is essential to guide management, it may be constrained by the temporal overlap of haemodynamic events. In clinical practice, the optimal approach integrates goal-directed haemodynamic correction with continuous bedside assessment of tissue perfusion. Pharmacological selection should account for the interaction between myocardial contractility and vascular tone, prioritising agents with combined or complementary effects. In this context, clinical judgement grounded in a sound understanding of pathophysiology remains the decisive tool in the management of mixed shock.

## RESUMEN

El shock mixto representa una entidad hemodinámica compleja resultante de la combinación variable de disfunción contráctil y vasoplejía. Su reconocimiento clínico es desafiante, ya que puede presentarse como una condición intermedia en la evolución del shock cardiogénico o distributivo, o como resultado de mecanismos fisiopatológicos simultáneos. La activación neurohormonal, común a ambos extremos del espectro, contribuye a la retención hídrica y al agravamiento de la disfunción circulatoria. La interpretación fisiopatológica, aunque esencial para orientar el tratamiento, puede verse limitada por la superposición temporal de los eventos hemodinámicos. En la práctica, el abordaje óptimo combina la corrección dirigida por objetivos hemodinámicos con la valoración clínica continua de la perfusión tisular. La elección farmacológica debe considerar la interacción entre contractilidad y tono vascular, priorizando agentes con efectos integrados. En este contexto, el juicio clínico fundamentado en la comprensión fisiopatológica sigue siendo la herramienta decisiva en el manejo del shock mixto.

## Introduction

The Shock Academic Research Consortium has recognised mixed shock as a distinct clinical phenotype within contemporary definitions of cardiogenic shock. In multicentre registries from cardiac intensive care units, approximately 17% of patients with shock meet criteria for mixed shock. This phenotype is associated with a higher likelihood of presenting in advanced stages of the SCAI shock classification (stages D or E), higher scores on the SOFA score, increased requirements for vasoactive medications, and often a greater burden of comorbidities. In addition, in-hospital mortality is higher in patients with mixed shock compared with other subtypes of cardiogenic shock ^1^.

**Figure 1:**
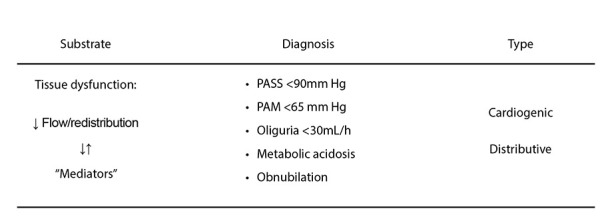
The definition includes the two variants of shock considered in this update: cardiogenic (myocardial depression) and distributive (vasoplegia). Tissue dysfunction results from reduced blood flow (systemic and redistribution) and the action of mediators (cytokines and endotoxins). ↓↑ indicates the interaction at the tissue level between reduced flow and mediators.

From a conceptual standpoint, shock is defined as tissue dysfunction secondary to reduced effective blood flow, either of cardiac origin or as a consequence of peripheral mediator activation ^2^. In many clinical scenarios, both mechanisms coexist and interact ^3^ (Figure 1).

Reduced tissue perfusion may result from decreased or redistributed cardiac output, reduced regional perfusion pressure, metabolic alterations induced by endotoxins or inflammatory mediators, or a combination of these factors ^3^.

This review focuses on circulatory failure resulting from cardiac pump failure or vasoplegia, particularly when both conditions coexist, giving rise to so-called mixed shock (MS) ^3^. Other aetiologies, such as hypovolaemic or neurogenic shock, may progress to severe circulatory failure that ultimately manifests with features of these phenotypes.

## Haemodynamics of mixed shock

**Figure 2:**
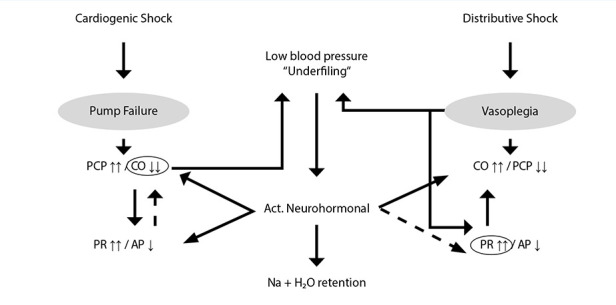
In cardiogenic shock, impaired cardiac function (central condition) reduces cardiac output (CO ↓↓) and increases pulmonary capillary pressure (PCP ↑↑). Neurohormonal activation results from arterial hypotension, relative hypovolaemia (underfilling), and reduced renal perfusion, leading to increased peripheral resistance (PR ↑↑) with sodium and wáter retention (Na + H₂O). This sequence constitutes a positive feedback mechanism that promotes progression of pump failure. In distributive shock, endotoxins (in sepsis) and inflammatory mediators induce profound vasoplegia with decreased peripheral resistance (PR ↓↓), resulting hypotension, and underfilling. Neurohormonal activation, together with reduced afterload, increases cardiac output (CO ↑↑). The dashed line indicates that neurohormonal activation does not counteract the vasodilatory effects of mediators. The reduction in arterial pressure may be partially compensated (AP ↓) by compensatory mechanisms: increased PR in cardiogenic shock and increased CO in distributive shock.

Figure 2 illustrates the pathophysiological sequences initiated by the primary haemodynamic disturbance (cardiac failure or vasoplegia) and the subsequent compensatory mechanisms.

In myocardial depression, the reduction in cardiac output triggers a compensatory neurohormonal response characterised by increased inotropy, heart rate, and volume retention. In this context, heart failure is interpreted by baroreceptors as relative hypovolaemia (underfilling), generating a positive feedback loop that further aggravates ventricular dysfunction ^2^^,^^4^^,^^5^.

In distributive shock, the initial event is vasoplegia secondary to endotoxins and inflammatory mediators. The resulting decrease in peripheral vascular resistance and hypotension is again perceived as relative hypovolaemia, leading to neurohormonal activation with similar downstream effects ^6^^,^^7^.

Neurohormonal activation does not correct contractile depression in cardiogenic shock nor vasoplegia in distributive shock; however, in both settings, it promotes sodium and water retention (Figure 2). Thus, underfilling reflects the perceived reduction in effective arterial filling, whether due to decreased cardiac output or vasoplegia, detected by vascular receptors.

**Figure 3:**
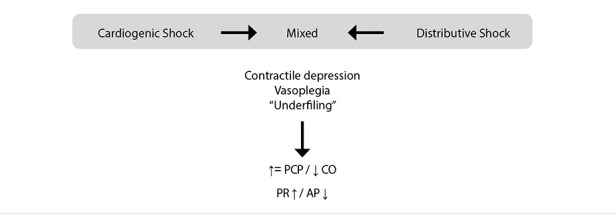
Haemodynamic failure in mixed shock (MS) results from the balance between mechanisms activated in cardiogenic shock (CS) and distributive shock (DS). Arrows indicate that MS may arise from CS following recovery of pump failure or from DS when myocardial function becomes impaired. See text for details.

MS represents a variable combination of contractile dysfunction and vasoplegia, resulting in an intermediate haemodynamic profile in which certain variables may appear normal depending on the relative contribution of each component (Figure 3).

PCP: pulmonary capillary pressure. CO: cardiac output. PR: peripheral resistance. AP: arterial pressure.

In this context, several reviews have addressed septic cardiomyopathy, a condition associated with complex pathophysiological mechanisms, including mitochondrial dysfunction, impairment of the myocardial microcirculation, and direct myocardial depression mediated primarily by proinflammatory cytokines and nitric oxide ^8^.

## Mixed shock as an intermediate condition

The term “mixed” implies that the haemodynamic state results from the coexistence, in variable proportions, of myocardial depression and vasoplegia ^2^. However, this definition can be expanded when considering the temporal evolution of the condition. From this perspective, MS may represent an intermediate stage in the progression of both cardiogenic and distributive shock (Figure 3).

In the first scenario, following the initial myocardial insult (e.g., ischaemia), vasoplegia may emerge during the reperfusion phase as part of the myocardial recovery process. Conversely, progression of distributive shock, with or without pre-existing heart disease, may lead to ventricular dysfunction and multiorgan failure, also resulting in a mixed haemodynamic state.

## Clinical scenario and pathophysiology

The pathophysiological interpretation, critical in the clinical approach to MS, requires addressing several key questions.

Is this a septic condition? If the answer is affirmative, the following considerations arise:

- Does the patient have no evident heart disease or a low probability of subclinical cardiovascular disease due to the absence of vascular risk factors? If so, it is likely that sepsis, during its course, has led to multiorgan damage with secondary cardiac involvement.
- Or is the patient a cardiac patient with an infectious complication? In this case, the infection has likely complicated the course of the underlying cardiovascular disease, which was the initial reason for hospitalisation.

Is this MS without evidence of systemic sepsis? If so, the following questions should be considered:

- Was there a condition of ischaemia-reperfusion? In such cases, initial myocardial depression is often followed by activation of the inflammatory response.
- In the absence of the above condition and after thoroughly excluding sepsis, additional considerations include:

Is vasoplegia a consequence of the primary clinical condition, for example, due to tissue injury associated with myocardial necrosis?

Or is it caused by another factor, such as exposure to medical devices, for instance, multiple intravascular catheters?

Recently, Jacob Van Diepen *et al.* proposed a framework to standardise the diagnosis of this condition. According to this approach, after adequate fluid resuscitation, or in the presence of elevated filling pressures (central venous pressure 8-12 mmHg or pulmonary capillary wedge pressure 12-15 mmHg), a vasodilatory component is suggested by low or normal systemic vascular resistance (SVR) (e.g., <1000-1200 dyn·s/cm⁵) or a low SVR index (<2000-2400 dyn·s/cm⁵/m²). Similarly, in initially vasodilatory states, a reduced cardiac index (CI <2.5 L/min/m²) or low cardiac output (CO <6 L/min), despite haemodynamic optimisation, may indicate a significant cardiac contribution. When interpreting these parameters, it is essential to consider the effects of vasoactive agents on haemodynamic measurements. These proposed thresholds should be viewed as guiding criteria rather than absolute cut-offs for defining MS ^9^.

**Table 1:** Haemodynamic variables in the three types of shock: cardiogenic, mixed, and distributive

Variable	Cardiogenic	Mixed	Distributive
SBP (mmHg)	80-90	<80	90-100
Cold skin, mottling	Yes	Yes	No
Urine output (<30 mL/h)	Yes	Yes	Yes
HR (beats/min)	80-110	90-120	100-140
CI (L/min/m²)	≤2.2	≤2.2	>3.5
PCP (mmHg)	>15	>15	<12
Systemic vascular resistance (dyn·s/cm⁵/m²)	1400-2000	600-1000	300-800
Ejection fraction (%)	30-40	30-40	>50

The values represent averages and may vary according to severity. In mixed shock, variables depend on the relative contribution of cardiac failure and vasoplegia.

HR: heart rate. CI: cardiac index. SBP: systolic blood pressure. PCP: pulmonary capillary pressure.

It is evident that the temporal sequence is a critical element in the pathophysiological interpretation of MS. However, in clinical practice, establishing the precise evolution of haemodynamic events is often challenging, which has important implications for therapeutic decision-making.

## From pathophysiology to decision-making

A central question is whether treatment should be guided by pathophysiological interpretation or by the correction of specific clinical and haemodynamic variables. Although the former approach appears conceptually superior, its application may be complex and, at times, prone to error.

For instance, attempting to reverse vasoplegia with a pure vasoconstrictor (such as vasopressin) in the presence of myocardial depression may worsen ventricular dysfunction ^10^. Conversely, the use of inotropic agents with vasodilatory effects (such as dobutamine) may exacerbate hypotension in a setting of predominant vasoplegia ^2^^,^^6^.

**Figure 4:**
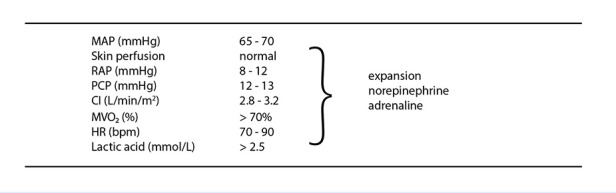
Haemodynamic targets to be achieved with pharmacological treatment.

Therefore, a more pragmatic strategy is to adjust haemodynamic variables towards target values and assess clinical response using parameters such as level of consciousness, urine output, skin perfusion, and trends in serum lactate. Figure 4 summarises commonly accepted haemodynamic targets ^2^.

The selection of noradrenaline, and, when appropriate, adrenaline, is based on their combined action on both components of mixed shock: myocardial depression and vasoplegia.

HR: heart rate. CI: cardiac index. RAP: right atrial pressure. MAP: mean arterial pressure. PCP: pulmonary capillary pressure. MVO₂: mixed venous oxygen saturation

Ideally, pathophysiological interpretation and goal-directed monitoring should converge. However, this theoretical alignment is often the result of retrospective analysis rather than a true dynamic integration of both approaches in real time.

Regarding therapeutic interventions, preload management is a critical component, always considering the concept of relative hypovolaemia. Among pharmacological options for MS, noradrenaline, and to a lesser extent adrenaline, are appropriate first-line agents due to their combined effects on myocardial contractility and vascular tone ^10^.

Other agents such as milrinone, dobutamine, levosimendan, or vasopressin may be used as adjunctive therapies, although they differ significantly in their effects on vasoplegia ^10^.

## Conclusion

An often underappreciated aspect in clinical practice is the pretest factor, that is, how the clinical context shapes both the interpretation of the condition and the therapeutic strategy. In general intensive care units, MS is more commonly associated with a predominance of vasoplegia, whereas in coronary care units myocardial depression is more frequently observed.

Moreover, patients admitted to cardiovascular units tend to be older and have a higher burden of vascular disease, factors that influence both the underlying pathophysiology and the response to treatment.

Ultimately, most available interventions exert mixed effects on myocardial contractility and vascular tone. Therefore, clinical judgement, grounded in pathophysiological understanding and continuous patient reassessment, remains the key determinant in the management of MS.
